# Does Serum 25-Hydroxyvitamin D Influence Muscle Development during Puberty in Girls? - A 7-Year Longitudinal Study

**DOI:** 10.1371/journal.pone.0082124

**Published:** 2013-12-16

**Authors:** Ru Wang, Markku Alen, Zhusheng Yu, Petri Wiklund, Shu Mei Cheng, Timo Törmäkangas, Peijie Chen, Sulin Cheng

**Affiliations:** 1 Key Laboratory of Exercise and Health Sciences of Ministry of Education at Shanghai University of Sport, Shanghai, China; 2 Department of Health Sciences, University of Jyväskylä, Jyväskylä, Finland; 3 Department of Medical Rehabilitation, Oulu University Hospital, Oulu, Finland; 4 Institute of Health Sciences, University of Oulu, Oulu, Finland; University of Tennessee, United States of America

## Abstract

Vitamin D is well known for its regulatory role in calcium and phosphate homeostasis, but its role in muscle mass and strength during growth remains inconclusive. We explored the association of serum 25-hydroxyvitamin D (25(OH)D) with muscle development in girls from 11 to 18-years old. Whole body lean tissue mass (LM_WB_), appendicular lean mass (aLM), muscle cross-sectional area at the lower leg (mCSA), maximal voluntary contraction of elbow flexors (MVC_elbow_) and knee extensors (MVC_knee_) were assessed in 217 girls aged 10–13 years (at baseline), 215 in 2-year and 226 in 7.5-year follow-up. Serum concentration of 25(OH)D and intact parathyroid hormone (PTH) were analyzed retrospectively and girls were categorized according to their 25(OH)D levels (consistently insufficient 25(OH)D G_LL_ <50 nmol/l and consistently sufficient G_HH_ >50 nmol/l from baseline to 7-year follow-up). We found that 25(OH)D level declined until menarche (p<0.05) while LM_WB_, aLM, mCSA, MVC_elbow_ and MVC_knee_ continued to increase (p<0.001 for all) post menarche. At pre-menarche, the G_LL_ (n = 34) had higher LM_WB_ and aLM than the G_HH_ (n = 21, p<0.05), while post-menarche the G_HH_ (n = 15) had a greater catch-up gain in LM_WB_ (p = 0.004), aLM (p = 0.001) and mCSA (p = 0.027) compared to the G_LL_ (n = 65) over the first 2-year period. At the age of 18, no differences in muscle mass/strength between the low (n = 151) and high (n = 77) levels of 25(OH)D groups were found. This finding was independent of vitamin D receptor genotype and other confounders. In conclusion, our results showed that levels of 25(OH)D have no significant negative influence on the development of muscle mass and strength during pubertal growth both with longitudinal and cross-sectional comparison. On the contrary, our results suggest that the temporary negative association between 25(OH)D and muscle mass arises as a consequence of fast growth prior to menarche, and this negative association is diminished through catch-up growth after menarche.

## Introduction

Recent epidemiological studies have shown that people with poor early growth have an increased risk of sarcopenia in later life, indicating that sarcopenia may have its origin during early development [1], [2]. Pubertal growth with optimal exercise and nutrition is considered to be a prime time for maximizing one’s genetically predetermined peak bone mass [3], but it is not clear if that is the case with muscle mass. It has been suggested that vitamin D has beneficial effects on the development and maintenance of muscle mass [4], [5]. The beneficial effects of serum 25 hydroxyvitamin D (25(OH)D) (an index of vitamin D status) are mediated by its receptor in skeletal muscle [6], [7], and genotypic variations for this receptor have been reported to be associated with differences in muscle strength [8], [9]. However, recent studies on the effects of 25(OH)D have primarily focused on elderly individuals who are most prone to sarcopenia [10], [11], [12]. Only a few studies have considered adolescents [13], [14]. These previous studies are cross-sectional in design and thus unable to examine whether the relationship between serum 25(OH)D level and muscle mass/strength is independent of growth and maturation [8]–[11], [13]–[15].

Puberty is a critical period of growth and development that is highly dependent on adequate nutrition. Vitamin D is well known for its regulatory role in calcium and phosphate homeostasis, and deficiency in vitamin D in childhood can cause reduced bone mineral density, delayed growth, skeletal deformities and impaired motor development [16]–[23]. However, the role of vitamin D in muscle mass and strength during growth is still inconclusive. In this study, we used a 7.5-year longitudinal dataset of pubertal girls to test our hypothesis that 1) a consistently low level of 25(OH)D has a significant negative influence on the development of muscle mass and strength in girls during the rapid pubertal growth period, and 2) the associations between level of 25(OH)D and muscle mass and strength gain are dependent on maturation stage and vitamin D genotypes.

## Subjects and Method

### Study Design

The subjects and the study design have been described previously [24], [25]. Briefly, 396 girls aged 10–13 years (mean age 11.2 years, at baseline) were recruited from local schools in the city of Jyväskylä and its surroundings in Central Finland to participate in a longitudinal study of determinants of body composition during puberty growth (the Calex-study). During the first 2-years, girls participated in a randomized intervention with calcium, vitamin D and dairy products supplementation. After the first 2-years intervention, we continued to follow the subjects on average for additional 5 years. The same data collections were performed at the 4- and 7.5-year time points. Because there were no intervention effects on muscle mass and strength the data were pooled together for the secondary analyses [24], [25].

### Study Population

Among the eligible participants who had both 25(OH)D and body composition assessments, 217 of them were included in the baseline, 215 in 2-year, 110 in 4-year and 226 in the 7.5-year follow-up (of those 226 girls, 101 had both baseline and 7.5-year follow-up assessments). At the 4-year follow, only part of the participants was randomly invited for body composition assessments due to limited funding. In other time point’s data collections, the main reasons for dropout were relocation, loss of interest and lack of time.

### Background Information

Current health status, chronic conditions and regular medications were checked by study nurse and physician. Dietary information was obtained from a food-intake diary kept for three days (two ordinary school days and one weekend day) as described elsewhere [26]. For this current report, the nutrient data related to body composition was used. Leisure time physical activity was assessed by questionnaire in terms of times/week and hours/week of participation in exercise. The age at menarche was defined as the first onset of menstrual bleeding and was determined by questionnaire or phone call during the follow-up. Body weight and height were measured with subjects wearing light clothes and on bare feet.

### 25-Hydroxyvitamin D (25-OHD) and Parathyroid Hormone (PTH) Assessments

The laboratory tests performed within two weeks’ period during the same month of the year throughout the 7.5-year follow-up to avoid seasonal effects. Blood samples were taken in the morning between 7∶00 and 9∶00 after an overnight fast. Serum samples were stored at –70°C until analyzed. Serum 25(OH)D concentrations were measured by radioimmunoassay (Incstar Corporation, Stillwater, MN). The intra- and inter-assay CVs were 10% and 15%, respectively. The reference range for 25(OH)D was 25–120 nmol/L. Serum intact parathyroid hormone (PTH) concentrations were measured by using an immunoradiometric method (Nichols Institute, Juan San Capistrano, CA), with 10–65 pg/L as the reference range. The intra- and inter-assay CVs were 4% and 3%, respectively.

### Vitamin D Receptor Gene Polymorphisms

Genomic DNA was extracted and purified from EDTA blood samples using the QIAmp Blood Kit (Qiagen GmbH, Hilden Germany). TaqI polymorphism at exon 9 of the VDR gene, ApaI polymorphism at intron 8 of the VDR gene and a C to T variation located at the restriction enzyme FokI site at the translation start site of VDR gene were analyzed [27]. Capital (T, A, F) and small (t, a, f) letters refer to the absence and presence of the restriction site, respectively. Genotypes AA, TT, and FF represent homozygotes of frequent alleles; Aa, Tt, and Ff represent heterozygotes, and aa, tt, and ff represent homozygotes of infrequent alleles.

### Body Composition, Muscle Mass and Size Assessments

Body composition was measured by dual-energy X-ray absorptiometry (DXA, Prodigy; GE Lunar Corp., Madison, WI USA). The CV of two repeated measurements on the same day was on average 1.0% for whole body lean mass (LM_WB_) and 2.2% for fat mass (FM). Appendicular lean mass (aLM) was calculated as the sum of lean mass in arms and leg as the surrogate for muscle mass.

In addition, the left lower leg was scanned using peripheral quantitative computerized tomography (XCT 2000, Stratec Medizintechnik, Pforzheim, Germany). The scan location was at 60% of the lower leg length up from the lateral malleolus of the fibula. The in-plane pixel size was 0.59 mm × 0.59 mm. The muscle cross-sectional area (mCSA) was analyzed using validated software (Geanie 2.1, Geanie®CE, Commit Ltd, Espoo, Finland). A contour was drawn manually along the outer boundary of muscle to eliminate the subcutaneous adipose tissue before analysis. The threshold for muscle was 10–279 mg/cm^3^. The CV of two repeated measurements on the same subject on the same day was on average 1% for mCSA.

### Muscle Strength and Performance Assessments

Maximal isometric voluntary contraction force (MVC, N) of the left elbow flexors and left leg extensors was measured in a sitting position with an adjustable dynamometer chair (Good Strength, Metitur, Jyväskylä, Finland). Subjects were encouraged to exert their maximal effort during an isometric test for ∼3 s. The MVC of the elbow flexors was measured with the subject’s forearm strapped at the wrist using velcro straps with elbow at 90° and the thumb in an upward position. Seat height was adjusted to ensure that the upper arm was at a 90° to the chest and the elbow was at the pivotal point of the apparatus. In the measurement of MVC of knee extensors, the subject’s lower leg was strapped at the distal end of the lower leg using Velcro straps with knee at 120°. Seat height was adjusted to ensure that the hip was at 90° to the trunk and the knee was at the pivot point of the apparatus. The hip was also strapped by Velcro straps to limit its movement. The CV was 3.7% and 6.6% for elbow flexors and knee extensors, respectively.

### Ethics

The study procedures followed were in accordance with the Helsinki Declaration of 1975 as revised in 1983. The study protocol was approved by the ethical committee of the University of Jyväskylä, the Central Hospital of Central Finland and the Finnish National Agency of Medicines (memo 1/2005, 19.01.2005, 3/2006, 5/4/2006, 20/2/2007, 18/12/2007, 22/4/2008, 6/6/2008, and 22/8/2008). The participants provided their written consent in accordance with the guidelines laid down by the ethical committees. When the girls were under the age of 18 years old, both participants and their parents signed the informed consent prior to the assessments.

### Statistical Analyses

All data were checked for normality using Shapiro-Wilk test in IBM SPSS 20.0 for Windows. A hierarchical (multilevel) model with random effects (ML wiN 2.22 software, Institute of Education, University of London, UK) was used to explore the patterns of longitudinal changes of 25(OH)D and muscle mass and strength during growth. The hierarchical model allows inclusion of the data from every subject regardless of irregularity of temporally spaced follow-up or missing data. Time relative to menarche (TRM), instead of age, was entered as the explanatory variable in the form of polynomial functions to explain the evolution of target variables.

The associations between longitudinal changes of muscle parameters and changes of 25(OH)D pre- and post-menarche were assessed by the following hierarchical model:
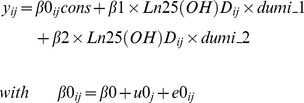



where the outcome variable *y_ij_* is LM_WB_, aLM, MVC_elbow_ and MVC_knee,_. The subscript indices represent the *i*th measurement occasion for the *j*th individual, respectively. The predictor variable is Ln(25(OH)D). β_0_ is the intercept, and both u_0j_ and e_0ij_ form the random portion of the model, whose means are equal to zero. The terms *dumi*_1 and *dumi*_2 are the dummy variables introduced to denote the pre- and post-menarche periods respectively. Here, the time of menarche itself is selected as a shift knot for the model, which means that the coefficients could be different on either side of this time point. Thus, the associations between 25(OH)D and muscle mass and strength can be assessed by regression coefficients β1 for pre-menarche, and by β2 for post-menarche, respectively.

In order to investigate whether the level of 25(OH)D had a significant influence on the development of muscle mass and strength during pubertal growth, we categorized girls according to their 25(OH)D levels and compared the two groups: G_LL_ = 25(OH)D consistently lower than 50 nmol/l both at baseline and 7.5-year follow-up, and G_HH_ = 25(OH)D consistently higher than 50 nmol/l both at baseline and 7.5-year follow-up. T-test was used to compare the two 25(OH)D groups.

In order to evaluate whether maturation status has influence on the relationship between 25(OH)D and development of muscle mass/strength, we used a mixed model to compare the percentage changes between G_LL_ and G_HH_ and between pre- and post-menarcheal girls controlled for age at the 2-year follow-up, and the percentage changes of body height, PTH, intakes of vitamin D and physical activity level. If there was overall significance (i.e. interaction between vitamin D group and maturation stage by measurement time points), pair-wise comparisons between vitamin D groups were assessed by the Šidák method within pre- and post-menarche. The results are reported as estimated means ± standard error (SEs). A P value of <0.05 was considered statistically significant.

Analysis of variance (ANOVA) was used to compare the group differences in VDR genotypes. In addition, when comparing the group differences in muscle mass and strength VDR Apal alleles were controlled for and P-values for the pairwise comparisons within pre- and post-menarche analyses were made using the Šidák method.

## Results

At the age of 11, 13 and 18 years, the proportion of subjects with vitamin D insufficiency [(25(OH)D ≥25 nmol/l and <50 nmol/l] was 56.5% and 59.3%, 63.4% and vitamin D deficiency [(25(OH)D <25 nmol/l] 6.0%, 12.6%, and 8.9%, respectively.

**Figure 1:**
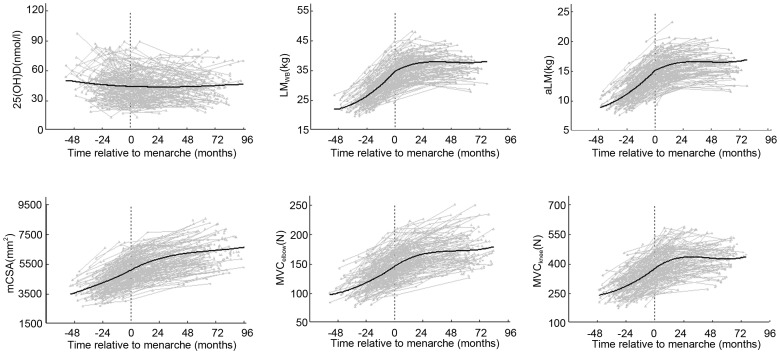
The growth curves of 25(OH)D (*a*), lean mass(*b* = LM_WB_ & *c* = aLM), muscle size (*d* = mCSA) and muscle strength (*e* = MVC_elbow_ & *f* = MVC_knee_). TRM = time (months) relative to menarche; LM_WB_ = whole body lean mass; aLM = appendicular lean mass; mCSA = muscle cross-sectional area; MVC_elbow_ = maximum voluntary muscle contraction of elbow flexors; MVC_knee_ = maximum voluntary muscle contraction of knee extensors. Grey dots and lines indicate individual values and the black solid line indicates the best fitting of growth pattern by hierarchical modelling.

The hierarchical model with random effects for longitudinal changes of serum 25(OH)D, LM_WB_, aLM, mCSA, MVC_elbow_ and MVC_knee_ in girls are presented in **Figure 1** **.** The concentration of 25(OH)D declined till menarche (p<0.05) but not post-menarche. LM_WB_, aLM, mCSA, MVC_elbow_ and MVC_knee_ continue to increase after menarche (p<0.001 for all).

We found that during pre-menarche 25(OH)D was negatively correlated with LM_WB_ (β = − 2.048 (SE = 0.477), p<0.05), aLM (β = −1.468 (SE = 0.388), p<0.01), mCSA (β = −0.323 (SE = 0.097, p<0.01 ), MVC_elbow_ (β = −0.041 (SE = 0.019), p<0.05), and MVC_knee_ (β = −0.050 (SE = 0.026), p>0.05), while at post-menarche, these negative associations disappeared (LM_WB_ (β = 0.196 (SE = 0.484), p>0.05), aLM (β = 0.705 (SE = 0.394), p>0.05), mCSA (β = 0.047 (SE = 0.099), p>0.05) MVC_elbow_ (β = 0.030 (SE = 0.020), p>0.05), MVC_knee_ (β = 0.025 (SE = 0.026), p>0.05).

Menarche is a critical time point for the development of girls. In order to evaluate whether maturation status has influence on the relationship between 25(OH)D and muscle mass/strength, we divided girls into pre- and post-menarcheal status at the first 2-year follow-up and compared the G_LL_ and G_HH_ groups. We found that the G_LL_ was more mature as reflected by TRM (p = 0.033), and taller (p = 0.034), and had lower PTH level compared to the G_HH_ (p = 0.0 28, **Table 1**). No other differences in terms of level of physical activity and dietary intakes of micronutrients between the G_LL_ and G_HH_ groups were found. Further, there were no significant differences in terms of distribution of different VDR genotypes of TaqI and FokI between the G_LL_ (TT/Tt/tt = 41/49/10% and FF/Ff/ff = 28/64/8%, respectively) and the G_HH_ (TT/Tt/tt = 42/50/8% and FF/Ff/ff = 17/75/8%, respectively) groups. The G_LL_ tended to differ from G_HH_ in ApaI (AA/Aa/aa G_LL_ = 26/64/10% vs. G_HH_ = 55/27/18%, p = 0.09). Controlling for VDR Apal, the G_LL_ group had higher LM_WB_ (p = 0.02), aLM (p = 0.021) than G_HH_ while no significant differences were found in mCSA, MVC_elbow_ and MVC_knee_ in pre-menarcheal girls but these differences were not present in post-menarcheal girls (**Table 2**).

**Table 1:** Comparison of anthropometry, physical activity, intake of micronutrients, and PTH among girls grouped according to whether their 25-OHD levels were consistently sufficient, insufficient, increased or decreased over the first 2-years follow-up, according to their menarcheal status (ANOVA; mean and SE are given, adjusted for multiple comparisons by the Šidák method).

Variables	At the 2-year follow-up pre-menarche		at the 2-year follow-up post-menarche	
	G_LL_ (n = 34)	G_HH_ (n = 21)	G_LL_ (n = 65)	G_HH_ (n = 15)
Age (years)	12.6 (0.1)	12.8 (0.1)	13.6 (0.1)	13.0 (0.2)
TRM (months)	−8.7 (1.4)	−11.8 (1.9)	10.1 (1.0)	6.2 (1.9)*
Height (cm)	154.9 (1.0)	150.7 (1.3)*	160.3 (0.8)	158.8 (1.3)
Weight (kg)	46.9 (1.7)	42.8 (2.2)	53.4 (1.3)	52.4 (2.6)
BMI (kg/m2)	19.4 (0.6)	18.7 (0.8)	20.8 (0.4)	20.8 (0.9)
FM (kg)	12.6 (1.2)	11.5 (1.5)	14.7 (0.9)	14.3 (1.8)
Exercise(times/wk)	3.00 (0.4)	3.54 (0.5)	3.06 (0.3)	3.38 (0.5)
Exercise (hrs/wk)	2.66 (0.4)	3.15 (0.5)	3.02 (0.3)	3.73 (0.6)
Intakes of Calcium (mg/d)	969 (81)	1068 (100)	1041 (59)	1057 (118)
Phosphorus (mg/d)	1205 (66)	1243 (81)	1210 (48)	1270 (96)
Vitamin D (µg/d)	3.01 (0.4)	4.15 (0.5)	2.0 (0.3)	4.37 (0.6)**
Energy (kcal/d)	1586 (176)	1769 (136)	1899 (90)	1567 (176)
Protein (E%)	15.2 (0.6)	15.6 (0.8)	15.3 (0.5)	15.5 (0.9)
Fat (E%)	31.6 (1.0)	34.4 (1.3)	33.2 (0.7)	30.6 (1.5)
Carbohydrate (E%)	53.2 (1.3)	49.9 (1.6)	51.5 (0.9)	53.9 (1.9)
Serum PTH(pg/ml)	32.1 (1.9)	27.4 (2.5)	30.3 (1.4)	24.2 (2.9)*

− TRM is pre- and +TRM is post-menarche. TRM = Time (months) relative to menarche; BMI = weight/height^2^; FM = fat mass. Pre- and post-menarche was divided by TRM value of the 2-year-follow-up time point where menarche is 0,

_LL_ and G_HH_: *<0.05; **<0.01. Significant difference between the G

**Table 2:** Comparison of lean mass and muscle strength between girls whose 25-OHD levels were consistently sufficient and consistently insufficient, according the menarcheal status at 2-year follow-up controlled for VDR Apal (ANOVA; mean and SE are given, adjusted for multiple comparison by the Šidák method).

	Pre-menarche		Post-menarche	
	G_LL_ (n = 34)	G_HH_ (n = 21)	G_LL_ (n = 65)	G_HH_ (n = 15)
LM_WB_ (kg)	33.0 (0.69)	29.4 (1.06)*	36.3 (0.5)	35.8 (1.05)
aLM (kg)	14.2 (0.35)	12.8 (0.54)*	15.7 (0.25)	15.7 (0.53)
mCSA (cm^2^)	48.4 (1.64)	44.9 (2.54)	55.4 (1.20)	56.2 (2.51)
MVC_elbow_ (N)	141.2 (4.5)	135.8 (7.0)	154.6 (3.3)	162.0 (6.9)
MVC_knee_ (N)	351.8 (14.2)	353.3 (22.0)	399.4 (10.3)	432.4 (21.7)

_WB_ = whole body lean mass; aLM = appendicular lean mass; mCSA = muscle cross-sectional area; MVC_elbow_ = maximum strength of elbow flexors; MVC_knee_ = maximum strength of knee extensors. LM

_LL_ and G_HH_. <0.05 pairwise comparisons between G

In addition, no significant differences were found between the G_LL_ and G_HH_ in any of the variables except for 25(OH)D level when girls at the age of 18 years. Further, including whole cross-sectional sample who participated in 7.5-year follow-up tests (n = 226), the results remained the same (**Table 3**).

**Table 3:** Comparison of muscle mass and strength among girls grouped according to whether their 25-OHD levels were sufficient and insufficient at the 7.5-years follow-up (T-test; mean and SD are given).

Variables	G_Low_ (n = 151)	G_High_ (n = 77)
LM_WB_ (kg)	38.2 (4.1)	37.8 (3.9)
aLM (kg)	17.0 (2.3)	16.5 (2.1)
mCSA (mm2)	6418 (1085)	6212 (1028)
MVC_elbow_ (N)	165 (30)	169 (40)
MVC_knee_ (N)	388 (93)	386 (81)

**Figure 2:**
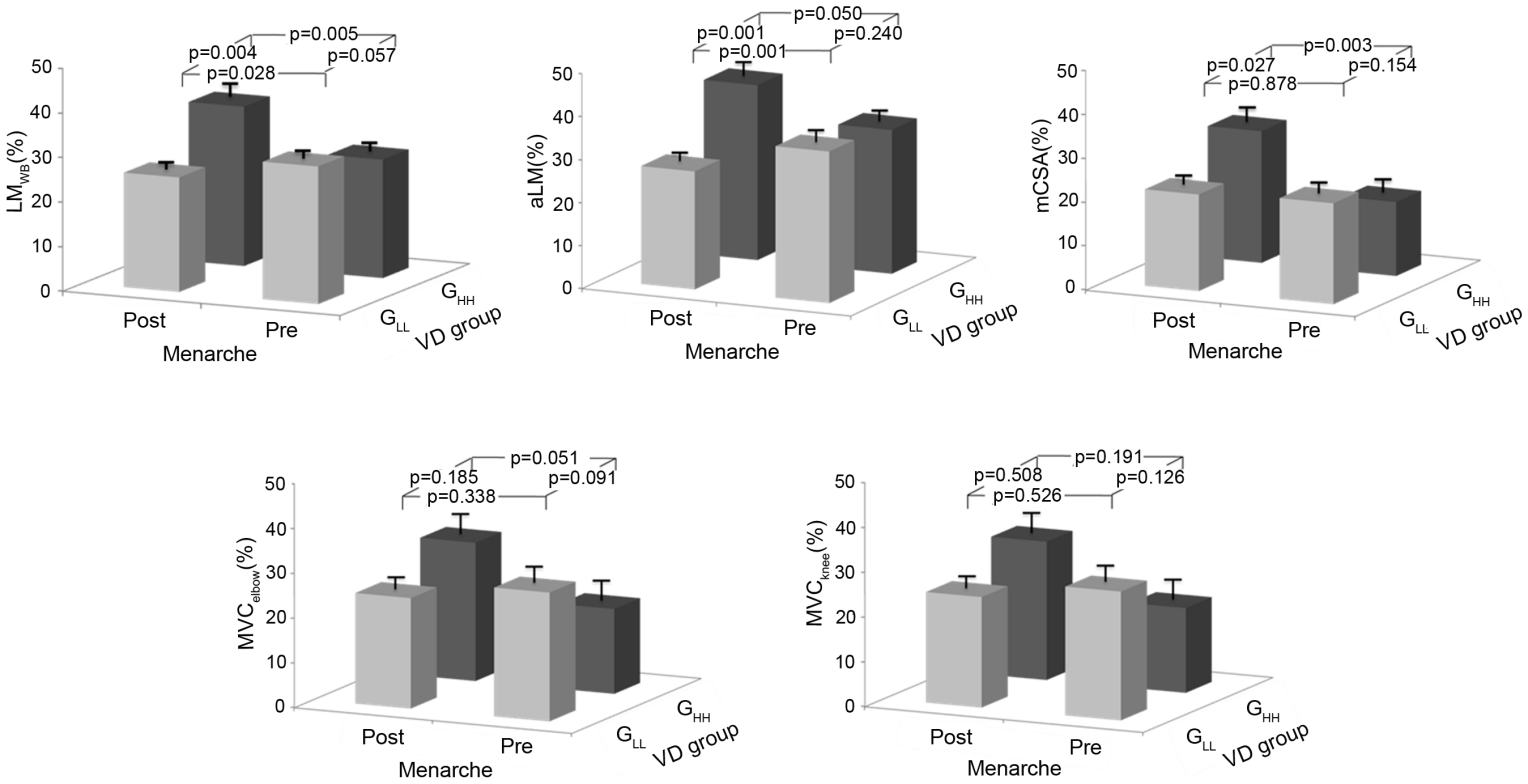
Comparison of changes of lean mass (*a* = LM_WB_ & *b* = aLM), muscle cross-sectional area (*c* = mCSA) and muscle strength (*d* = MVC_elbow_ & *e* = MVC_knee_) during the 2-year period in pre- and post-menarche girls at the 2-year follow-up time point. Estimated mean with SE (error line) controlled for age and change of body height, vitamin D intakes, level of physical activity and PTH. LM_WB_ = whole body lean mass; aLM = appendicular lean mass; mCSA = muscle cross-sectional area; MVC_elbow_ = maximum voluntary muscle contraction of elbow flexors; MVC_knee_ = maximum voluntary muscle contraction of knee extensors; G_HH_ = consistently vitamin D sufficient; and G_LL_ = consistently vitamin D insufficient.

We further compared the percentage change of the muscle mass and strength between the G_LL_ and G_HH_ groups during the first 2-year period, controlling for those confounding factors which may influence 25(OH)D and muscle mass and strength development (age, and the changes of body height, vitamin D intakes, level of physical activity and PTH). We further compared the percentage change of the muscle mass and strength between the G_LL_ and G_HH_ groups during the first 2-year period, controlling for those confounding factors which may influence 25(OH)D and muscle mass and strength development (age, and the changes of body height, vitamin D intakes, level of physical activity and PTH). We found that, there was an interaction between the menarche status, pre- vs. post-groups, and the vitamin D status, sufficient vs. insufficient groups, in change of LM_WB_ (p = 0.001), aLM (p = 0.003) and mCSA (p = 0.009). By contrast to the G_LL_, the G_HH_ group increased more in LM_WB_ (p = 0.004, **Figure 2a**), aLM (p = 0.001 **Figure 2b**) and mCSA (p = 0.027 **Figure 2c**) post-menarche. Within the G_HH_ group, they increased more in LM_WB_ (p = 0.005, **Figure 2a**) and mCSA (p = 0.003 **Figure 2c**) in post-menarche than pre-menarche, while within the G_LL_ group, they increased more in LM_WB_ (p = 0.028, **Figure 2a**) and aLM (p = 0.001 **Figure 2b**) during pre-menarche than post-menarche. No significant differences between the vitamin D sufficient and insufficient groups were found in the percent change of MVC_elbow_ (Figure 2d) and MVC_knee_ (**Figure 2e**). However, the G_HH_ group tended to increase more their MVC_elbow_ post-menarche compared to the G_LL_ group (p = 0.091) and pre-menarche (p = 0.051).

We found that girls with Apal allele of AA had higher LM_WB_, aLM and mCSA than their counterparts with Aa and aa alleles at the baseline, 2-year and 7.5-year follow ups (**Table S1**). After controlling for the level of 25(OH)D the results remained the same.

## Discussion

Our data demonstrate that, during growth, there was a temporary negative association between 25(OH)D and muscle mass and strength development prior to menarche due to fast growth. After menarche these inverse associations disappeared. At the age of 18 years, no differences in muscle mass or strength between the 25(OH)D groups were found at the 7.5-year follow-up.

Vitamin D status of children in transition through puberty is an important area of study, especially at latitudes above 37 degrees north wherein the cutaneous synthesis of vitamin D is only possible for no more than half the year [16]–[18]. The high prevalence of 25(OH)D insufficiency (<50 nmol/l) in our study is consistent with earlier reports showing that 25(OH)D insufficiency is common in growing Finnish children and adolescents [19], [20]. Similarly, a number of studies worldwide, even in sunny areas [21], have reported a high prevalence of 25(OH)D insufficiency in healthy (asymptomatic) growing children and adolescents [22], [28]–[30]. The high prevalence of 25(OH)D insufficiency during childhood is alarming and calls for additional research to establish a level of 25(OH)D that optimizes skeletal development during growth and prevents potential negative health effects later in life.

Although the role of vitamin D in skeletal health and development has been a hot topic, the impact of vitamin D on muscle mass and function during growing period is less clear. Cross-sectional studies have reported positive relationship between 25(OH)D, and forearm muscle (grip) strength [31] and jump height, velocity and power [32] in adolescent girls. El-Hajj et al. [33] showed in a randomized controlled study that vitamin D3 (cholecalciferol) supplementation significantly increased lean mass in 10–17 years old females. Whereas another prospective study showed an age-related decline in serum 25(OH)D level in girls aged 4–8 years over a period of 1–7 years [32]. The authors found that the decline of 25(OH)D level with age was eliminated after controlling for LM, suggesting that the increase in LM during growth is associated with a decline in circulating 25(OH)D concentration. Further, Ward et al. found no improvements in muscle force or power with one year vitamin D2 (ergocalciferol) supplementation in postmenarchal 12- to 14-yr-old girls [34]. Goswami et al. in turn showed that while 6 months cholecalciferol/calcium supplementation effectively increased and maintained serum 25(OH)D, the increase did not improve skeletal muscle strength in young girls [35]. Our data show that the low LM_WB_ and aLM, MVC_elbow_ and MVC_knee_ at the baseline in the vitamin D sufficient group disappeared at 2-year follow-up indicating that the associations between 25(OH)D and muscle mass and strength is maturation stage dependent. Moreover, the temporary negative association between 25(OH)D) and muscle mass and strength prior to pre-menarche does not have long-term effects on development of muscle mass and strength as indicated by the fact that no differences in muscle mass or strength between groups were found when girls were at the age of 18 years.

Vitamin D receptor (VDR) polymorphisms have been suggested to be associated with muscle strength, but evidence for this is limited and conflicting [36]. In our study, no significant differences in terms of distribution of different VDR genotypes between groups were found. However, we found that the girls with VDR Apal AA homozygotes of frequent alleles had higher LM_WB_, aLM and mCSA than their counterparts with Aa and aa alleles at 2 and 7.5-year follow-up, suggesting that certain VDR polymorphisms may affect muscle mass development in girls during growing period. The molecular basis for the association between VDR Apal polymorphism and muscle mass in the present study remains unclear, and more research is needed to confirm and explain these associations.

Vitamin D intakes and other lifestyle factors have also been shown to influence both level of 25(OH)D [37] and muscle mass and strength in children [38] and old women [39]. We found no significant differences between the vitamin D insufficient and sufficient groups in terms of physical activity and micronutrient intake except for the intakes of vitamin D: the insufficient group had less consumption of vitamin D than the sufficient group in post-menarche. However, the intake of Vitamin D was not associated with the level of 25(OH)D and the change of muscle mass was independent of change in vitamin D intakes in this study.

PTH may also have a direct effect on skeletal muscle because PTH has been shown to impair energy production, transfer and utilization in skeletal muscle in rats [40] and influences skeletal muscle protein and amino acid metabolism in rats [41]. PTH is also known to increase free intracellular calcium concentrations in muscle tissue [42] which may disrupt muscle structure or function [43]. However, only a few studies have shown that PTH may influence muscle mass and strength, independent of either 25(OH)D or 1,25(OH)2D levels [44], [45]. Our data showed that high levels of PTH were associated with low levels of 25(OH)D. However, the level of PTH was not associated with muscle mass and strength gain and the relationship between 25(OH)D and muscle mass was independent of PTH.

### Strengths and Limitations of Study

Despite several strengths of our study, including the longitudinal data followed girls from pre-puberty to early adulthood and use of hierarchical (multilevel) model in which allowed us to evaluate the change of 25(OH)D and muscle mass/strength during the entire growth period. The results of our study must be interpreted in the light of its limitations. First, few subjects in our cohort had very low levels of 25(OH)D (especially at baseline, 6% **<**25 nmol/l), limiting statistical power to detect an effect of severe vitamin D deficiency. Second, tissue levels of vitamin D may be more important with respect to muscle mass or strength than the serum levels assessed here, although there is now controversy about whether skeletal muscle even possesses receptors for vitamin D [46]. Thirdly, participants were Finnish girls, and the degrees to which genetics, and latitude, or other environmental factors may have contributed to these differences remain unclear, hence the results may not necessarily apply to other populations. Furthermore, analyses were adjusted for multiple factors, but the possibility of residual confounding factors cannot be excluded. Finally, although most of relevant research suggests that circulating 25(OH)D is recognized as the best functional indicator of vitamin D status, 1,25(OH)_2_D was not assessed and this must be considered as a limitation. Focusing only on serum 25(OH)D, information on the functional role of vitamin D during peripubertal muscle growth may not have been captured.

## Conclusion

Our results showed that when comparing the levels of serum 25(OH)D between the two groups with lower to higher than 50 nmol/l, no significant negative influence of 25(OH)D on the development of muscle mass and strength during pubertal growth were found both with longitudinal and cross-sectional comparison. On the contrary, our results suggest that the temporary negative association between 25(OH)D and muscle mass arises as a consequence of fast growth prior to menarche, and that this negative association is diminished through catch-up growth after menarche.

## Supporting Information

Table S1 **Comparison of 25(OH)D, muscle mass and strength among different genotypes in girls at the baseline, 2-year and 7-year follow-up (ANOVA by Šidák method for multiple comparison; quoted values are mean and SD).** LMWB = whole body lean mass; aLM = appendicular lean mass; mCSA = muscle cross-sectional area; MVCelbow = maximum strength of elbow flexors; MVCknee = maximum strength of knee extensors. *<0.05 pairwise comparisons between AA and Aa, and AA and aa.
(DOCX)
