# Supplementary material for: Does Serum 25-Hydroxyvitamin D Influence Muscle Development during Puberty in Girls? - A 7-Year Longitudinal Study
Source: PLoS One. 2013 Dec 16;8(12):e82124. doi: 10.1371/journal.pone.0082124 (PMC3864869; doi:10.1371/journal.pone.0082124)
Supplement: Table S1 — Comparison of 25(OH)D, muscle mass and strength among different genotypes in girls at the baseline, 2-year and 7-year follow-up (ANOVA by Šidák method for multiple comparison; quoted values are mean and SD). LMWB = whole body lean mass; aLM = appendicular lean mass; mCSA = muscle cross-sectional area; MVCelbow = maximum strength of elbow flexors; MVCknee = maximum strength of knee extensors. *<0.05 pairwise comparisons between AA and Aa, and AA and aa. (DOCX) [file pone.0082124.s001.docx]

Table S 1.

|  | **Apal** | | | | **Fok** | | | | **Taq** | | |
| --- | --- | --- | --- | --- | --- | --- | --- | --- | --- | --- | --- |
| **Baseline** | **AA (n=78)** | **Aa (n=121)** | **aa (n=46)** | **FF (n=103)** | | **Ff (115)** | **ff (n=29)** | **TT (106)** | | **Tt (n=118)** | **tt (n=23)** |
| 25(OH)D (nmol/l) | 50.3 (15.4) | 44.1 (15.2)* | 42.9 (13.5)* | 44.7 (14.9) | | 47.3 (15.7) | 45.4 (14.3) | 43.2 (13.9) | | 47.7 (15.6) | 50.2 (17.4) |
| LM_WB_ (kg) | 27.8 (4.3) | 27.0 (4.3) | 26.6 (3.9) | 26.9 (4.4) | | 27.4 (4.0) | 27.3 (4.4) | 27.1 (4.3) | | 27.1 (4.0) | 28.0 (4.8) |
| aLM (kg) | 11.7 (2.2) | 11.4 (2.1) | 11.2 (1.9) | 11.3 (2.1) | | 11.6 (2.1) | 11.5 (2.3) | 11.5 (2.1) | | 11.4 (2.0) | 11.9 (2.8) |
| mCSA (cm^2^) | 44.7 (8.7) | 42.3 (8.1) | 41.5 (8.2) | 43.0 (8.5) | | 42.7 (8.1) | 43.9 (8.7) | 42.4 (8.1) | | 43.0 (8.1) | 45.1 (10.1) |
| MVC_elbow_ (N) | 126 (20.3) | 121 (23.1) | 120 (23.0) | 122 (20.1) | | 122 (23.2) | 127 (26.5) | 122 (22.9) | | 122 (21.9) | 126 (22.8) |
| MVC_knee_ (N) | 310 (65.2) | 290 (64.4) | 280 (62.4)* | 295 (61.5) | | 293 (67.8) | 308 (70.9) | 291 (62.1) | | 299 (67.5) | 297 (72.0) |
| **2-year** | **AA (n=69)** | **Aa (n=108)** | **aa (n=41)** | **FF (n=95)** | | **Ff (98)** | **ff (n=26)** | **TT (95)** | | **Tt (n=103)** | **tt (n=21)** |
| 25(OH)D (nmol/l) | 41.6 (15.0) | 42.8 (17.6) | 41.5 (17.0) | 41.4 (17.2) | | 43.4 (16.1) | 41.3 (16.5) | 42.7 (16.3) | | 42.1 (17.2) | 41.6 (15.7) |
| LM_WB_ (kg) | 35.8 (4.4) | 34.0 (4.6)* | 33.8 (4.1) | 34.8 (4.6) | | 34.3 (4.3) | 34.5 (5.0) | 34.2 (4.5) | | 34.6 (4.4) | 35.8 (4.8) |
| aLM (kg) | 15.6 (2.2) | 14.7 (2.2)* | 14.6 (2.1) | 15.1 (2.2) | | 14.9 (2.2) | 15.9 (2.7) | 14.8 (2.1) | | 15.0 (2.3) | 15.7 (2.6) |
| mCSA (cm^2^) | 55.5 (10.5) | 50.4 (9.3)** | 50.8 (9.4)* | 53.2 (10.0) | | 50.9 (9.5) | 52.9 (11.3) | 51.1 (9.2) | | 52.1 (9.9) | 56.9 (12.3) |
| MVC_elbow_ (N) | 154 (22.9) | 149 (27.6) | 149 (24.2) | 153 (25.3) | | 149 (24.8) | 148 (30.1) | 151 (25.3) | | 150 (26.5) | 155 (23.4) |
| MVC_knee_ (N) | 403 (78.4) | 388 (81.1) | 376 (82.9) | 392 (76.0) | | 390 (84.0) | 389 (87.6) | 385 (83.6) | | 394 (77.9) | 399 (83.4) |
| **7-year** | **AA (n=34)** | **Aa (n=50)** | **aa (n=14)** | **FF (n=36)** | | **Ff (n=54)** | **ff (n=9)** | **TT (38)** | | **Tt (n=53)** | **tt (n=9)** |
| 25(OH)D (nmol/l) | 43.4 (13.8) | 43.0 (15.8) | 46.0 (15.5) | 41.1 (15.2) | | 45.3 (15.0) | 43.4 (12.5) | 43.9 (16.8) | | 44.8 (13.5) | 35.9 (12.6) |
| LM_WB_ (kg) | 38.7 (3.6) | 36.8 (4.3) | 35.2 (3.7)* | 37.8 (4.3) | | 37.2 (4.1) | 36.0 (3.9) | 37.5 (4.5) | | 37.3 (4.2) | 36.3 (1.6) |
| aLM (kg) | 17.1 (2.1) | 16.1 (2.2) | 15.5 (2.1)* | 16.6 (2.1) | | 16.4 (2.4) | 15.8 (2.3) | 16.4 (2.3) | | 16.6 (2.3) | 15.6 (1.6) |
| mCSA (cm^2^) | 65.9 (10.2) | 59.7 (9.7)* | 58.2 (7.8)* | 62.8 (10.5) | | 61.2 (11.0) | 62.0 (4.8) | 60.4 (9.5) | | 62.6 (10.9) | 63.5 (10.6) |
| MVC_elbow_ (N) | 188 (29.9) | 178 (31.9) | 167 (23.3) | 183 (28.3) | | 181 (31.7) | 168 (33.8) | 178 (27.6) | | 181 (34.4) | 183 (19.8) |
| MVC_knee_ (N) | 422 (80.9) | 430 (98.2) | 390 (77.2) | 431 (90.7) | | 422 (79.3) | 379 (132) | 432 (75.9) | | 418 (94.6) | 395 (116) |
